# Supplementary material for: Causal reasoning without mechanism
Source: PLoS One. 2022 May 13;17(5):e0268219. doi: 10.1371/journal.pone.0268219 (PMC9106179; doi:10.1371/journal.pone.0268219)
Supplement: S1 Appendix — Norming data for study 4a and 4b. (DOCX) [file pone.0268219.s001.docx]

List of items used in Study 1

| quartz watch  cylinder lock  zipper  piano  flush toilet  sewing machine  can opener  water faucet  self-winding watch  aqualung  snare  ball-point pen  spray-bottle  fireplace | solid-fuel rocket  incinerator  greenhouse  nuclear reactor  steam central heating  car ignition system  VCR  speedometer  35mm camera  LCD screen  cellular phone  fluorescent light  car gearbox  electric motor | helicopter  manual clutch  hydroelectric turbine  jet engine  car differential  landline telephone  car battery  microchip  ethernet network  transistor  computer mouse  scanner  photocopier  television |
| --- | --- | --- |

Table A1. Norming data for study 4a and 4b (M: mechanical; C: chemical; E: electromagnetic).

| *Item* | *Likelihood (M%) (SD%)* | | *Intended*  *Domain* | *Proportion M* | *Proportion C* | *Proportion E* | *X^2^* | *p* | *> ^1^/_3 agreement_* |
| --- | --- | --- | --- | --- | --- | --- | --- | --- | --- |
| A random person had to have a limb cut off, or amputated. | 46.45 | 39.30 | M | .74 | .19 | .06 | 73.87 | .000 | Yes |
| A random person suffered physical trauma after being involved in a head-on car accident. | 81.57 | 24.56 | M | .70 | .20 | .10 | 18.60 | .000 | Yes |
| A random person suffered from a blood disease. | 61.00 | 35.97 | C | .07 | .90 | .03 | 43.40 | .000 | Yes |
| A random person's car wasn't very shiny after treating it with wax. | 51.55 | 32.71 | C | .10 | .90 | .00 | 139.17 | .000 | Yes |
| A random person used the wrong king of wax when waxing their car. | 54.27 | 31.83 | C | .33 | .63 | .03 | 16.20 | .000 | Yes |
| A random person used the wrong waxing motion when waxing their car. | 62.50 | 33.68 | M | .87 | .03 | .10 | 38.60 | .000 | Yes |
| A random person's sweater got a hole in it. | 74.86 | 26.57 | M | .86 | .11 | .03 | 118.87 | .000 | Yes |
| A random person spilled bleach on their sweater. | 54.50 | 34.63 | C | .20 | .80 | .00 | 31.20 | .000 | Yes |
| A random person's sweater got caught on their belt's fastener. | 58.77 | 31.62 | M | .93 | .03 | .03 | 48.60 | .000 | Yes |
| *A random statue has started to slowly break down.* | *71.13* | *30.36* | *M* | *.15* | *.81* | *.04* | *97.11* | *.000* | *No* |
| *A random coal-burning plant operated near a statue.* | *41.23* | *31.69* | *C* | *.27* | *.47* | *.27* | *2.40* | *.301* | *Yes* |
| *A random dam operated near a statue.* | *50.77* | *32.98* | *M* | *.73* | *.07* | *.20* | *22.40* | *.000* | *Yes* |
| Most of the urine in a random super absorbent diaper was absorbed. | 82.10 | 18.92 | M | .36 | .64 | .00 | 57.79 | .000 | Yes |
| A random super absorbent diaper contains lots of tiny pores that suck up liquid. | 72.93 | 31.29 | M | .57 | .43 | .00 | 15.80 | .000 | Yes |
| A random super absorbent diaper contains chemicals that react with liquid to transform the liquid. | 50.13 | 36.58 | C | .03 | .97 | .00 | 54.20 | .000 | Yes |
| *The timing on a random person’s quartz wristwatch is inaccurate.* | *61.40* | *31.16* | *E* | *.79* | *.02* | *.19* | *91.23* | *.000* | *No* |
| *A random person shook their quartz wristwatch violently.* | *49.70* | *31.70* | *M* | *.80* | *.03* | *.17* | *30.20* | *0.000* | *Yes* |
| *A loudspeaker played music near where a random person left their wristwatch.* | *46.23* | *33.91* | *E* | *.47* | *.00* | *.53* | *15.20* | *.001* | *Yes* |
| The fan's inside a random person's laptop suddenly slowed down. | 66.28 | 30.31 | M | .72 | .01 | .27 | 73.55 | .000 | Yes |
| A random person's laptop was kept in a very dusty room. | 67.13 | 30.57 | M | .87 | .00 | .13 | 39.20 | .000 | Yes |
| A random person's wireless network cut off while streaming a movie. | 72.93 | 28.77 | E | .10 | .00 | .90 | 43.80 | .000 | Yes |
| A random ceiling fan stopped working. | 60.28 | 33.35 | M | .74 | .00 | .26 | 80.77 | .000 | Yes |
| The rotor inside a random fan broke. | 51.03 | 30.70 | M | .83 | .00 | .17 | 35.00 | .000 | Yes |
| The power source feeding a random fan broke. | 49.27 | 35.48 | E | .20 | .03 | .77 | 26.60 | .000 | Yes |
| A random person got sick after using a dirty restroom. | 56.71 | 33.26 | C | .06 | .91 | .02 | 143.32 | .000 | Yes |
| A random person rubbed their hands together under running water when cleaning their hands. | 89.73 | 22.69 | M | .60 | .13 | .27 | 10.40 | .006 | Yes |
| A random person submerged and left their hands in soapy water before rinsing them off when cleaning their hands. | 59.30 | 32.76 | C | .57 | .40 | .03 | 13.40 | .001 | Yes |
| A person put white craft glue on two random light-weight pieces of wood. Usually they stick together well, but this time they didn't. | 46.60 | 31.24 | C | .15 | .84 | .01 | 111.47 | .000 | Yes |
| A couple of random pieces of wood were finely sanded. | 64.10 | 33.59 | M | .87 | .07 | .07 | 38.40 | .000 | Yes |
| A couple of random pieces of wood were chemically-treated for weather. | 61.50 | 32.24 | C | .00 | 1.00 | .00 | 60.00 | .000 | Yes |
| A random person's house caught on fire. | 55.13 | 38.40 | C | .12 | .48 | .40 | 20.57 | .000 | Yes |
| A random fireplace was lit with a wool sweater nearby. | 34.00 | 33.34 | C | .27 | .50 | .23 | 3.80 | .150 | Yes |
| A random person plugged their air conditioner into an extension cord. | 60.97 | 31.88 | E | .30 | .00 | .70 | 22.20 | .000 | Yes |
| A random person found a hammer in a bucket of milky-looking water. The hammer was no longer rusty. | 37.09 | 32.95 | C | .02 | .98 | .00 | 176.26 | .000 | Yes |
| A random bottle of sodium chloride spilled into a mixture, which then sat overnight. | 32.00 | 30.89 | C | .00 | 1.00 | .00 | 60.00 | .000 | Yes |
| A live electrical contact came into contact with a random hammer. | 37.10 | 32.57 | E | .17 | .03 | .80 | 30.20 | .000 | Yes |
| *After laying out in the sun, a random person's skin turned red and started to blister.* | *76.71* | *27.18* | *C* | *.02* | *.47* | *.51* | *41.45* | *.000* | *Yes* |
| *A random person drank a gallon of tomato juice before tanning.* | *16.17* | *22.91* | *C* | *.47* | *.43* | *.10* | *7.40* | *.025* | *Yes* |
| *A random person wore dark sunglasses to block the sun while tanning.* | *84.43* | *23.54* | *E* | *.47* | *.23* | *.30* | *2.60* | *.273* | *no* |
| A random person's cell phone battery doesn't hold charge as well as it used to. | 84.26 | 22.30 | E | .21 | .24 | .54 | 18.66 | .000 | Yes |
| A random person phone's vibrated during incoming calls. | 83.00 | 21.50 | M | .53 | .03 | .43 | 12.60 | .002 | Yes |
| A random person phone's battery never fully drained. | 58.73 | 37.56 | E | .23 | .23 | .53 | 5.40 | .067 | Yes |
| The radio in a random car no longer works. | 57.70 | 34.52 | E | .69 | .01 | .30 | 65.89 | .000 | Yes |
| A random car was in an accident. | 82.53 | 23.95 | M | .97 | .00 | .03 | 54.20 | .000 | Yes |
| A random car's battery short-circuited. | 54.90 | 35.36 | E | .10 | .10 | .80 | 29.40 | .000 | Yes |
| *A random person owned a CD that wouldn't play right, but now it does.* | *53.56* | *33.95* | *E* | *.84* | *.02* | *.14* | *110.70* | *.000* | *No* |
| *A random person accidentally smeared toothpaste on their CD.* | *22.43* | *26.79* | *C* | *.67* | *.27* | *.07* | *16.80* | *.000* | *No* |
| *A random person accidentally left their CD in the freezer overnight.* | *15.70* | *22.46* | *E* | *.63* | *.20* | *.17* | *12.20* | *.002* | *No* |
| *A random person’s credit card didn’t swipe properly, but now it does.* | *66.38* | *30.46* | *E* | *.71* | *.05* | *.23* | *65.51* | *.000* | *No* |
| *A random person covered their credit card’s magnetic strip with tape.* | *27.60* | *28.38* | *M* | *.60* | *.00* | *.40* | *16.80* | *.000* | *Yes* |
| *A random person demagnetizing the magnet in the latch on their wallet.* | *35.43* | *31.46* | *E* | *.17* | *.07* | *.77* | *25.80* | *.000* | *Yes* |
| A random person reheating a dish in the microwave found it to be less warm than usual. | 67.81 | 28.58 | E | .15 | .07 | .78 | 83.89 | .000 | Yes |
| A random person substituted butter for oil when preparing a meal. | 76.17 | 26.95 | C | .33 | .60 | .07 | 12.80 | .002 | Yes |
| A random person overloaded the circuit their microwave was on. | 56.90 | 37.53 | E | .03 | .03 | .93 | 48.60 | .000 | Yes |
| A random person making bread found their loaf to be smaller than they hoped. | 69.98 | 26.69 | C | .24 | .68 | .07 | 55.17 | .000 | Yes |
| A random person didn't knead the dough enough when making bread. | 65.67 | 32.48 | M | .57 | .40 | .03 | 13.40 | .001 | Yes |
| A random person used too much yeast when making bread. | 64.70 | 29.35 | C | .10 | .87 | .03 | 38.60 | .000 | Yes |
