# Supplementary material for: Causal reasoning without mechanism
Source: PLoS One. 2022 May 13;17(5):e0268219. doi: 10.1371/journal.pone.0268219 (PMC9106179; doi:10.1371/journal.pone.0268219)
Supplement: S2 Appendix — Norming data for Study 5. (DOCX) [file pone.0268219.s002.docx]

Table A2. Norming data for Study 5 (M: mechanical; C: Chemical; E: electromagnetic)

| *Item* | *Likeli-*  *hood (M%)* | *Likeli-*  *hood (SD%)* | *Intended*  *Domain* | *Proportion M* | *Proportion C* | *Proportion E* | *X^2^* | *p* | *> ^1^/_3 agreement_* |
| --- | --- | --- | --- | --- | --- | --- | --- | --- | --- |
| A person's house lost power. | 77.06 | 31.77 | E | 0.40 | 0.02 | 0.58 | 24.52 | 0.000 | Yes |
| A person's house caught on fire. | 59.26 | 39.84 | C | 0.28 | 0.60 | 0.12 | 17.92 | 0.000 | Yes |
| A person left their wool sweater too close to a lit fireplace. | 48.88 | 40.42 | C | 0.30 | 0.60 | 0.10 | 19.00 | 0.000 | Yes |
| A person plugged their air conditioner into an extension cord. | 64.58 | 33.90 | E | 0.36 | 0.02 | 0.62 | 27.16 | 0.000 | Yes |
| *A meal came out dryer than usual.* | *67.40* | *34.18* | *C* | *0.32* | *0.54* | *0.14* | *12.04* | *0.002* | *Yes* |
| *A meal came out colder than usual.* | *71.30* | *31.90* | *E* | *0.48* | *0.36* | *0.16* | *7.84* | *0.020* | *No* |
| *Before microwaving a meal, a person substituted butter for oil in it.* | *62.22* | *36.43* | *C* | *0.20* | *0.76* | *0.04* | *42.88* | *0.000* | *Yes* |
| *The microwave used to prepare a meal was plugged into an overloaded circuit.* | *58.28* | *35.47* | *E* | *0.22* | *0.00* | *0.78* | *48.52* | *0.000* | *Yes* |
| The alignment in a car does not work right. | 66.26 | 34.94 | M | 1.00 | 0.00 | 0.00 | 100.00 | 0.000 | Yes |
| The stereo in a car does not work right. | 66.84 | 36.86 | E | 0.58 | 0.02 | 0.40 | 24.52 | 0.000 | Yes |
| A car was in an accident. | 72.08 | 33.69 | M | 1.00 | 0.00 | 0.00 | 100.00 | 0.000 | Yes |
| A car was shocked by a downed power line. | 45.74 | 40.96 | E | 0.14 | 0.00 | 0.86 | 63.88 | 0.000 | Yes |
| *The battery in a person's quartz watch stopped working right.* | *69.10* | *34.96* | *E* | *0.34* | *0.22* | *0.44* | *3.64* | *0.162* | *Yes* |
| *The timing on a person's quartz watch stopped working right.* | *59.50* | *37.64* | *M* | *0.64* | *0.06* | *0.30* | *25.48* | *0.000* | *No* |
| *A person left their quartz watch on top of a speaker.* | *51.24* | *37.15* | *E* | *0.36* | *0.04* | *0.60* | *23.68* | *0.000* | *Yes* |
| *A person violently shook their quartz watch.* | *51.06* | *37.83* | *M* | *0.90* | *0.04* | *0.06* | *72.28* | *0.000* | *Yes* |
| The reception on a cell phone no longer worked right. | 68.90 | 35.04 | E | 0.24 | 0.00 | 0.76 | 45.28 | 0.000 | Yes |
| The vibrator on a cell phone no longer worked right. | 62.40 | 38.85 | M | 0.66 | 0.00 | 0.34 | 32.68 | 0.000 | Yes |
| A person dropped their phone. | 82.88 | 28.37 | M | 0.94 | 0.02 | 0.04 | 82.84 | 0.000 | Yes |
| A person left their phone near a powerful magnet. | 46.80 | 39.70 | E | 0.06 | 0.04 | 0.90 | 72.28 | 0.000 | Yes |
| A person used white craft glue to join two pieces of wood together, but they didn't line up very well. | 66.48 | 34.59 | M | 0.68 | 0.30 | 0.02 | 32.92 | 0.000 | Yes |
| A person used white craft glue to join two pieces of wood together, but they didn't stick very well. | 65.62 | 37.17 | C | 0.26 | 0.70 | 0.04 | 33.88 | 0.000 | Yes |
| Some pieces of wood were finely sanded. | 72.04 | 34.85 | M | 0.86 | 0.12 | 0.02 | 63.16 | 0.000 | Yes |
| Some pieces of wood were chemically-treated for weather. | 68.24 | 35.97 | C | 0.02 | 0.98 | 0.00 | 94.12 | 0.000 | Yes |
| A person's sweater got discolored. | 69.40 | 33.37 | C | 0.06 | 0.90 | 0.04 | 72.28 | 0.000 | Yes |
| A person's sweater got holes in it. | 72.28 | 33.09 | M | 0.66 | 0.30 | 0.04 | 29.08 | 0.000 | Yes |
| A person poured bleach over a stain on their sweater. | 54.60 | 36.90 | C | 0.04 | 0.94 | 0.02 | 82.84 | 0.000 | Yes |
| A person rubbed steel wool over a stain on their sweater. | 32.68 | 37.97 | M | 0.60 | 0.30 | 0.10 | 19.00 | 0.000 | Yes |
