# Supplementary material for: Causal reasoning without mechanism
Source: PLoS One. 2022 May 13;17(5):e0268219. doi: 10.1371/journal.pone.0268219 (PMC9106179; doi:10.1371/journal.pone.0268219)
Supplement: Attachment — Submitted filename: Response to reviewers.docx [file pone.0268219.s004.docx]

*We thank the reviewers for their comments and greatly appreciate the feedback as these will help to straightening the manuscript. We respond below in turn to the questions raised by each reviewer; our comments are in italic.*

**Reviewer #1 (R1)**

This paper examines a longstanding puzzle in cognitive psychology: On the one hand, many results point to the importance of mechanism knowledge in causal reasoning; yet on the other hand, people are remarkably bad at articulating these mechanisms, suggesting that mechanism knowledge is shallow and skeletal. This paper presents evidence for a domain-matching heuristic, such that people categorize causal mechanisms into broad domains (e.g., mechanical, chemical, electromagnetic), matching causes and effects by domain despite a lack of detailed knowledge of how these mechanisms work. Several experiments in both adults and children support this hypothesis.

Overall, I enjoyed reading this paper and think it makes a contribution to the literature on causal cognition. Here are a few questions I had while reading it, mainly aimed at further understanding the authors’ theory and how it fits in with other perspectives.

*Thank you*

1. The coverage in the introduction of the literature on mechanism knowledge in causal reasoning is generally very good. For readers who are unfamiliar with phenomena such has the illusion of explanatory depth and the related results from the authors’ own research group, it would be useful to devote a couple more paragraphs to summarizing the evidence that people have impoverished knowledge of causal mechanisms.

*We have added text to the first couple of paragraphs to fulfil the reviewer’s request.*

2. One relevant literature the authors do not consider is related work on other similarity-matching heuristics in causal reasoning. Off-hand, I can think of at least four papers in this vein that the authors might find useful in terms of explaining and situating their theory:

Einhorn, H. J., & Hogarth, R. M. (1986). Judging probable cause. Psychological Bulletin, 99(1), 3-19.

Johnson, S. G. B., & Keil, F. C. (2014). Causal inference and the hierarchical structure of experience. Journal of Experimental Psychology: General, 143(6), 2223.

LeBoeuf, R. A., & Norton, M. I. (2012). Consequence-cause matching: Looking to the consequences of events to infer their causes. Journal of Consumer Research, 39(1), 128-141.

Lim, J. B., & Oppenheimer, D. M. (2020). Explanatory preferences for complexity matching. PloS one, 15(4), e0230929.

Work on the “laws of sympathetic magic” is also broadly relevant, although less directly about casual reasoning in particular:

Rozin, P., Millman, L., & Nemeroff, C. (1986). Operation of the laws of sympathetic magic in disgust and other domains. Journal of Personality and Social Psychology, 50(4), 703–712.

It would be useful if the authors could explain how their similarity-matching principle relates to the ideas in these papers. Does it share a similar adaptive or informational logic?

*We have cited most of these papers and explained their relation to our hypothesis in multiple changes on p. 2-3.*

3. I am a little confused about the authors’ use of terminology surrounding “domains.” This is not entirely on the authors, as this terminology is somewhat slippery in the broader literature as well.

(i) *Categories* In a somewhat loose sense, sometimes people use the word “domain” to simply refer to different categories of knowledge or phenomena. For example, professors at a music conservatory might have different “domains” of knowledge such as music history, music theory, and music performance. In that sense, the categories studied in this paper (e.g., electromagnetism vs. mechanics) surely count as domains. It seems like there’s nothing inherently special about these particular categories, but rather these are the level of categories that participants naturally learned. In the vein of work by Coley, Medin, and others on expertise and categorization, these categories could easily shift with increased knowledge and likely would become more specific with more experience.

(ii) *Modules* In its more technical sense, “domain” refers to something more like a mental module. For example, folk-psychology or folk-physics could be a domain because core knowledge about these domains plausibly evolved, whereas folk-music-history would not be a good candidate for a domain, nor (I think) would folk-electromagnetism. Presumably modules (to the extent they exist) have not changed over the course of human history, but I am sure that everyday intuitions about electromagnetism have changed very much over the past 100 years.

As you can see, I think the authors are basically using the word “domain” synonymously with “category,” but this is causing me some confusion as there are some references to the domain-specificity literature that make it seem like the authors are sometimes using some of the theoretical apparatus of modules.

Related – and perhaps this would help with the above point – the discussion of Sloman, Lombrozo, and Malt’s analysis is not integrated as well as it could be with the other points made in the introduction. I like the Sloman et al. chapter a lot and find it a good way of summarizing various writers’ views on this topic, and I also find those authors’ “mild ontology” view plausible. But the relationship between mild ontology and the “domain matching” hypothesis isn’t particularly clear, mainly because “domain specificity” or “extra strong ontology” is very much along the lines of modules (option ii), whereas “mild ontology” is much more along the lines of categories (option i).

Basically, I would like the authors to better articulate what they mean by “mechanism domains,” and possibly consider using a clearer term that does not carry as much baggage and ambiguity.

*We thank the reviewer for this important point. We have modified the text to be clearer throughout the introduction, including this additional sentence on p. 4: “We refer to such types as “domains.” Our claim is not that mechanisms come in a fixed hierarchy of types, but that any appropriate abstraction process induces a category and therefore a domain.”*

4. One thing that made the paper a little bit hard to read was the introduction of alternative explanations throughout the presentation of the results rather than in the front-end of the paper. Could the authors add a section that collects the alternative accounts in one place (e.g., similarity, base rates, mechanism knowledge) and explains the kind of evidence that will be important for addressing them? These alternatives are not explained in very much detail in the current version of the manuscript which makes it somewhat difficult to evaluate their plausibility. In particular, the similarity account seems like a plausible alternative that should be discussed more thoroughly, both in the introduction and discussion, as fleshing this out would likely help to clarify the authors’ own proposal.

*We have tried to clarify alternative explanations throughout the paper. However, collecting them all in one place struck us as adding unduly to the length of the paper.*

5. Overall I found the package of studies to be convincing. I had just a couple suggestions around the presentation:

(i) The results of Study 1 sound very interesting but they are only described qualitatively. Could the authors provide some visualization of how participants sorted the items?

*Study 1 has been revised, and more figures have been added.*

(ii) Could the authors present figures that show the age effects (including comparisons to adults when the data are comparable)? Even though age effects were not substantial, the developmental aspect of these studies will be interesting to many readers.

*We have added the following figure to address this point (p. 27).*

**Fig 6.** Participants’ choices across age groups

Minor:

- The numbering of the tables is confusing, as the tables are numbered sequentially between the main text and appendix. Referring to the appendix tables as A1 and A2 (or simply as Appendix 1 and 2) would be clearer.

*Tables in appendixes have been named as A1, A2 and corrected in the manuscript as suggested.*

- Some of the references to tables seem to refer to the incorrect table, including lines 446, 474, and 521… generally throughout Studies 4 and 5 the relationship between the main text and tables is confusing.

*All the table numbers with their in-text references have been checked and corrected.*

Thanks very much for the opportunity to read your work, and best of luck with next steps!

*Thank you.*

**Reviewer #2 (R2)**

Review of “Causal reasoning without mechanism” by Dündar-Coecke, Goldin, and Sloman
In this paper, Dündar-Coecke, Goldin, and Sloman (DGS henceforth) investigate how reasoners (human adults and children) assess the causal status of co-occurred events in the absence of specific mechanistic or covariational knowledge about the corresponding types of events. The main hypothesis DGS propose in this paper is that people assess causal links between events in such cases by considering abstract mechanism domains. More specifically, the authors propose and test the idea that reasoners are more likely to regard certain events as causally connected if these events belong to the same global mechanism class rather than to different mechanism classes. The authors first established (Experiment 1) and then tested (subsequent studies) three global mechanism categories: (1) mechanical, (2) chemical, and (3) electromagnetic. For both adults and children, it was found that causal judgments, irrespective of whether they were made in the predictive or the diagnostic direction (Experiments 2 and 3), corresponded to the proposed domain matching hypothesis: when selecting the most probable cause (or effect) of a target event, subjects tended to select an event that belonged to the same mechanism category. Experiment 4 replicated the effect with test times (cause and effect events) that were more concrete than the rather abstract items used in Experiments 2 and 3.

I think that this is an interesting topic and that DGS propose a, in my view, very plausible hypothesis. I find it quite natural, for example, that if a reasoner observes an event and tries to uncover its cause(s), he or she will search for events that could plausibly be causally relevant. I share the authors’ intuition that broad mechanism categories are a useful guide in this respect. I also think that the question how people form causal beliefs in the absence of directly relevant information, such as information about covariation, has been understudied. However, although I’m quite enthusiastic about DGSs’ research, I think that some more work needs to be done before this manuscript can turn into an impactful publication. Some of my remarks, that can probably be dealt with fast, concern the flow and the presentation of ideas and conclusions, and the lacking of some information in the empirical sections, but I also think that an additional final experiment could be necessary to have a more convincing package.

*Thank you. We hope that following amendments address these concerns.*

R2: Flow of ideas and structuring:
My impression is that the main theory of the paper is told relatively straightforwardly, but I also was a bit confused by some of the paragraphs in the introduction and I think that many of the paragraphs are not very well connected. I fear that this tends to impair the reading flow. For example, in section 1.1 “Causal attribution in the absence of mechanism knowledge” it was not really clear to me what the authors wanted to tell the reader. If the purpose here was to convince the reader that reasoners tend to be lacking specific mechanism knowledge in many domains but often seem to be guided by a more abstract sense of mechanisms, that this could be done more concisely.

*Section 1.1. begins with a short description of the notion of mechanism. After that paragraph we revised the next paragraph as follows:*

*“Exposure to mechanisms is inevitable. Within months of taking our first steps, we start establishing some appreciation of how the world works without developing a deep understanding of operating mechanisms. Children learn to keep their ice-cream in the fridge on a sunny day. Cooks will use an oven to bake a cake. How exactly do people think they understand phenomena without knowing the details of causal interactions between functional parts? We argue that mechanism domains offer a heuristic for a variety of causal reasoning tasks so that even though most of us do not know how a fridge or an oven work, we rely on our beliefs that are rooted in knowledge of mechanism domains. It is this ability that helps us to organize knowledge into content domains like biology or chemistry.*

*Findings showing young children’s ability to distinguish animals from artifacts supports this view in the sense that children demonstrate distinct explanatory understandings of, for instance, biological (animals) and mechanical causal agents (machines or blocks). They seem to believe there are distinct mechanisms driving the causal relations in different domains [17], with multiple causal-explanatory construals for physical, biological, psychological, and chemical events. For instance, when asked, a preschooler could state that hammers break things, whereas water makes things wet, possibly by associating causes with effects in a domain-specific manner [18-21].”*

R2

I really wondered, for example, why the paragraph on page 5 beginning in line 108 was in there. The paragraph starts with “`The domain-based difference’ hypothesis, proposed by [17], suggests that domains can indeed play functional role even after equating strength of belief. In that study, a sample of people were asked several questions from the scientific and religious domains […]” and ends with “[…] This showed that people think a mechanistic explanation is a stronger requirement in science than religion.” I don’t understand why this is relevant for the present paper? I had the same question for the paragraph bout Rozenbit and Keil’s results starting in line 145, in which DGS summarize that people’s confidence in their understanding of devices decreases, for example, after they had tried to offer an explanations of how these devices work. Again, I think the point that DGS want to make in their theory section is that people do not seem to rely on specific mechanism knowledge, but this doesn’t mean that they are not guided by more abstract mechanism knowledge. I think the different paragraphs need to be connected better. Right now, it feels a bit like loose pieces and the main message emerges to the reader only somewhat implicitly.

*Yes, we agree with the reviewer on this point and have removed that paragraph accordingly.*

Presentation of empirical results:
I think it would be appropriate to give some rationale for the sample sizes that the authors employed in their studies. Did the authors do an a priori planning of their studies? Are the sample sizes based on pilot or previous studies?

*The sample sizes were based on a pilot work as well as the availability of resources. We also considered the minimum sample sizes necessary for the ANOVAs. This information was missing, we have added the following text in the paper (p.9):*

*“The sample sizes across the studies were based on a pilot work as well as the availability of volunteer participants. Using the G-Power software, minimum power to detect effect sizes in ANOVAs was .80 -a generally accepted level of power.”*

In the results sections when the authors present the means, I think it would be good to also report confidence intervals for the means and not just standard deviations. A related point is that, in Figure 2, the authors present 95% Cis for the proportions but SEs for the Likelihoods. Why? I think this should be the same for the bars in that figure. Since most readers will probably not expect different types of error bars within the same graph, a spontaneous impression (until one reads the legend) is that the proportion judgments were estimated much less precisely than the likelihood judgments, but this was actually not the case. Also, in Figure 1, the reader is not told what the error bars are. I think it would be best to have 95% CIs all the time.

*Figures 4 and 5 were revised, they both now contain error bars at 95% CIs without SE values.*

R2: Paragraph beginning in line 253:
DGS wrote “[…] their sorting conforms to the predicted mechanism domains: mechanical, chemical, and electromagnetic.” I think these domains were not predicted in the introduction. I was wondering how the authors can claim here that they found the predicted types of categories given that the goal of that study and the conducted cluster analysis was to identify relevant domains?

*That sentence has been corrected. It now reads as follows (p. 14):*

*“They not only suggest that participants are sensitive to mechanism, but also that their sorting conforms to three mechanism domains: mechanical, chemical, and electromagnetic.”*

R2: Items shown in Table 4:
I was wondering whether at least some of the items would have led to totally different results if they had been formulated slightly differently. Take, for example, Item 3: “Alfie made a bread, but he found it to be smaller than he hoped.” The different cause items are “Alfie didn’t mix the dough enough when making bread.” and “Alfie used too much yeast when making bread”. Subjects found the first option to be more likely, but what would have happened if the second option had mentioned “too little” rather than “too much” yeast. Using too little yeast leads to smaller breads, so wouldn’t this have changed subjects selections?

*There was a typo here. We meant to say too ‘little’ rather than too ‘much’. It has been corrected.*

The items shown in Table 4 bring me to my biggest concern, which I think might have to be addressed in a final separate study. While I think that the authors convincingly address the potential base rate problem, another problem that I see is that subjects might have thought about items and might have based their selections on considerations of causal strength. Take for example, Item 9 about hole in the sweater, which is supposed to be an event belonging to the mechanical domain. The two potential causes are that bleach was spilled on the sweater and (chemical cause) and the sweater got caught on her belt’s fastener (mechanical). Most subjects apparently chose the “belt fastener”. Apart from belonging to the same domain, doesn’t this cause also have a higher causal strength in leading to this effect? I think it is statistically more likely that a sweater carries away a hole if I it gets tangled up in the belt faster than when it gets treated with bleach. I think most people who use bleach will not use so much that holes results, while an accidental rip of a pullover on the belt fastener is not unlikely to lead to a hole. An even more drastic example is Item 1 about the square that doesn’t fit through a round hole. The potential causes are that the square was to large for the hole and that their colors didn’t match. Most people know that color is causally irrelevant, while size is not. This knowledge, I think, is very concrete and I don’t think subjects’ decisions here were guided only by means of abstract domain matching.
I think that DGSs’ results would be more convincing if they controlled for the causal strength between the potential causes and the effect. While controlling for base rate, I think that it might be a possibility that subjects might have based their decisions on causal strength considerations. I see that DGS aimed to address this concern in their Study 5 (intended to control for how “causative” one event is for the other), but I don’t think that the way they did this is compelling. The idea in Study 5 was to control for how “causative” causes are by pairing them with different effects. For example, the cause item “a person rubbed steel wool over their sweater” was once coupled with the effect item “A person’s sweater got discolored” and once with the effect item “A person’s sweater got holes in it”. Another alternative cause item that was also used was “A person poured bleach over a stain on their sweater”. In the main task, subjects were presented with one of the effect items and asked to select the most likely cause. It was found, for example, that subjects who saw the effect item “A person’s sweater got holes in it” tended to select the mechanical cause item “A person rubbed steel wool over their sweater”, while they tended to select the cause item “A person poured bleach over a strain on their sweater” when the effect item was “A person’s sweater got discolored”. My concern is that subjects here simply selected the causes that have a higher causal strength in generating the presented target effects. Spilling bleach over a sweater will rarely lead to a hole in a sweater, whereas the causal strength between “rubbing a sweater with steel wool” and “making a hole in the fabric” is probably much higher. The reverse is true for the “discoloration” effect. DGS write that if one of the causes is more “causative” then subjects’ preference shouldn’t change depending on the effect item. But causal strength is relative. One and the same cause can have high causal strength with respect to one type of effect and weak (or zero) causal strength with respect to another. I was also wondering whether a causal strength theory can actually be dissociated from the domain matching theory that DGS propose. If not, I think what would be needed if causal strength and domain matching always make similar predictions is an experiment that shows that subjects do not think of causal strength (although it would lead to the same behavior if they did) when searching for the likeliest cause, but really about broader mechanism classes.

*The reviewer is correct about these points, and that is the reason why we ran Study 5. In the last study, we particularly included multiple items to overcome this issue, as not all items clearly favoured causal strength. The causal strength of each cause for specific effects is higher when the causes match the domain of the effect. This is a possibility in principle. Our claim is that the reason for greater causal strength is because the cause and effect match domain. Perhaps each of our items suffers from a different confound, but the one invariant across items is that causal strength is higher when domains match. We take this as pretty good evidence that the reason that one cause has greater causal strength for one effect and not the other is because it shares a domain with the former.*

R2

Minor comments:
- Line 153: I think a paragraph shouldn’t start with “So…”

*It has been removed.*

- Line 153: “[…] gab between, on one hand, […]” should be “on the one hand”

*It has been corrected*

- Line 175: “This implies that mechanism domains are not parts of a […]” should be “part” not “parts” *This point has been addressed*

- Line 185: “Study 2 and 3 tested one […]” should be “Studies 2 and 3 tested one […]”

*This has been fixed.*

- Line 187: “[…], study 4 and 5 […]” should be “Studies 4 and 5 […]”

*This point has been addressed too.*

- Line 211: “[…] size and already constucted […]” should be “constructed”

*The typo has been corrected.*

- Line 313: “In the mechanical effect, the mechanical cause […]” should be sth. like “In the mechanical effect condition, […]”

*Yes, the sentence has been changed as suggested. It reads as follows (p.17): “In the mechanical effect condition […]”*

- Line 341: “[…], so we test the base rate account in Study 4.” Should be “tested”

*This has been addressed.*

- Line 488: “As predicted, people selected […]” should be “subjects”

*We replaced ‘people’ with ‘participants’ here and elsewhere where it was necessary.*

- Line 497: “[…] causes even if when […]” should be either “if” or “when”

*The sentence now reads as […] the within-domain causes when those causes […]*

In the reference list, many journal names were abbreviated “Cog Psyc.” or “Phil of Sci,” and I wondered whether this format is correct.

*The reference list has been prepared by following the PlosOne referencing style guidance (* [*https://journals.plos.org/plosone/s/submission-guidelines#loc-references*](https://journals.plos.org/plosone/s/submission-guidelines#loc-references)*). However, if reviewers think that this format is incorrect, we can, of course, revise it.*

All in all, I think that this is an interesting project and I think that this work can make an interesting contribution. However, I think that the writing requires some polishing; my main point here is that I think that the paragraphs are often to loosely connected. I also think that DGS need to address the causal strength argument, although I unfortunately can’t suggest a specific experiment right now that would address this issue straightforwardly.

 *Thank you.*

**Reviewer #3 (R3):**

This paper examines whether adults and children use a "mechanism domain" heuristic when connecting causes to effects. They find that people tend to associate independently-validated 'mechanical', 'chemical', and 'electromagentic' effects with causes in the same domain, and vice versa.

I have several concerns about Study 1, but I also believe that it could be either substantially expanded or removed from the paper and the end result would be suitable for publication in PLoS ONE.

First, the main issue with Study 1 is simply the lack of direct data available to readers, or at least reviewers. There is no listed repository that I could see linked in any of the materials or supplemental information that would let me see the raw data from this study, and the results are described at an extremely coarse level of detail. While the authors claim in the data availability statement that the data are available in the supporting information files, no information on Study 1 is found in either appendix, and no other files were in what I, as a reviewer, was able to download. At a minimum, I would have expected a table showing the individual items in each cluster in the mechanism condition (if not in each condition), since the identification of the clusters as "mechanical", "chemical", and "electromagnetic" is not currently supported in any way other than the author's assertion and a handful of examples from each cluster.

 *We hope that following amendments diminish these concerns.*

However, Study 1 may also be simply redundant, because each experiment includes its own stimulus validation experiment. Right now, with so little information about Study 1 available, I read the categories as largely arbitrarily defined, but that's not necessarily a problem given 1) the validation in each experiment and 2) the consistency of the results. While it would be nice to have a more complete version of Study 1 to give stronger external validation of the categories, the complexity of the analysis, and the underlying degrees of experimenter freedom about, for example, the cutoffs, mean that the paper may be most improved by simply omitting it and treat the three categories as being defined a priori. Since the stimuli of Studies 2-5 are not in fact based of the body of stimuli used in Study 1, Study 1 doesn't even provide direct evidence for these categories as they apply in the other experiments regardless.

 *We discussed the role of Study 1 and agreed with the reviewer that this study needs more elaborations. Accordingly, following amendments have been made:*

1. *The following paragraph has been added (p.10).*

*“The purpose of this study is to determine how people would sort randomly sampled items, whether they sort different set of real-world items in similar or dissimilar ways, that is, in such a manner that a hierarchical analysis could reveal the domains of mechanisms. To begin, we ran a study to enumerate the domains people use to think about common artifacts.”*

2. *A full list of items used in Study 1 has been added to Appendix 1 (p.10, line 232).*
3. *Three figures have been added to demonstrate how division of items was captured the mechanism domains (see Figures 1, 2, 3. Lines 264, 271, 279 respectively).*
4. *The following paragraph has been added to explain how similarities between mechanism, function, and general item clusters were compared (lines 280-302):*

*“In order to compare clusterings, three metrics were employed, namely the Rand Index (RI: Rand, 1971), the adjusted Rand Index (ARI: Hubert & Arabie, 1985), and the Variance of Information criterion (VI; Meila, 2007). These metrics’ results were shown in Table 1 below.”*

***Table 1.*** *Similarities between the mechanism, function, and general item clusterings (higher numbers mean greater similarity for the RI and ARI, while lower numbers mean greater similarity for the VI).*

|  | *Mechanism* | *Function* | *General* |
| --- | --- | --- | --- |
| *Mechanism* |  | *RI = 0.77*  *ARI = 0.51*  *VI = 0.64* | *RI = 0.72*  *ARI = 0.40*  *VI = 1.01* |
| *Function* |  |  | *RI = 0.85*  *ARI = 0.66*  *VI = 0.61* |

*The RI is derived by counting the number of times a given pair of items are grouped together or* *separately in two clusterings divided by these counts as well as the number of times they are grouped inconsistently (i.e., together in one clustering but separately in the other). The index spans from 0 to 1, whereby identical clusterings obtain 1 and clusterings that share no consistency score 0. The ARI spans the same range but is designed to be more sensitive to differences of that are the result of chance. The VI takes an information-theoretic approach, attempting to discern how much information one clustering can provide about the other. As such, two identical clusterings would obtain a VI score of 0 (i.e., no information can be gleaned about the other), and the VI has an upper bound of log n, where n is the number of points in the data set, since the more items there are, the more clusterings might theoretically exist.*

*Across each metric the function and general clusterings were similar, while the mechanism condition is a bit closer to the function than the general condition. These results offer preliminary support for our hypothesis. They not only suggest that people are sensitive to mechanism, but also that their sorting conforms to the predicted mechanism domains: mechanical, chemical, and electromagnetic.*

That said, studies 2-5 are excellent tests of the idea that people cluster mechanisms by domain. Study 5 in particular provides an elegant control for any concerns related to the individual stimuli. I believe that these studies fit all the criteria for publication in PLoS ONE, and either providing much more information about Study 1 or dropping it altogether will yield a paper that is suitable for publication.

*Thank you*

Minor things:

R3: Line 341: The base rate experiment is Study 3, not Study 4.

*This is corrected as “Study 3”*

R3: Line 453: "previous chapter" previous experiment?

*It has been corrected as “previous experiment”*
