# Supplementary material for: Causal reasoning without mechanism
Source: PLoS One. 2022 May 13;17(5):e0268219. doi: 10.1371/journal.pone.0268219 (PMC9106179; doi:10.1371/journal.pone.0268219)
Supplement: Attachment — Submitted filename: Response to reviewers2.docx [file pone.0268219.s005.docx]

*Once again, we thank the reviewers for their comments and greatly appreciate the feedback. We respond below the comments of the reviewer; our comments are in italic.*

**Reviewer #2 (R2)**

Reviewer #2: Review of “Causal reasoning without mechanism” #Revision 1 by Dündar-Coecke, Goldin, and Sloman

I’d like to apologize for the delayed submission of my review. I was Reviewer #2 in the first review round and now got the chance to read the revised manuscript.

My overall impression hasn’t changed. I think that this is very interesting work with the potential to make a relevant contribution to the literature on causal cognition.

I also think that the authors did an overall great job (though see below) in addressing the concerns and suggestions provided by the three reviewers. My two main former concerns were:

*Thank you.*

(1) “However, I think that the writing requires some polishing; my main point here is that I think that the paragraphs are often to loosely connected.

I think that the authors have addressed this concern very well.

(2) I also think that DGS need to address the causal strength argument, although I unfortunately can’t suggest a specific experiment right now that would address this issue straightforwardly.”

By contrast, I think that this second of my concerns has still not been resolved satisfactorily. My feelings that many of the thoughts the authors have provided in their reply (in the response letter) to this concern would be worth including in the discussion of the results, or even the general discussion. The authors in their reply wrote “The causal strength of each cause for specific effects is higher when the causes match the domain of the effect. This is a possibility in principle. Our claim is that the reason for greater causal strength is because the cause and effect match domain. Perhaps each of our items suffers from a different confound, but the one invariant across items is that causal strength is higher when domains match.”. If domain-matching and causal strength were to be prima facie confounded, shouldn’t this interesting observation be presented and discussed? The authors continued “We take this as pretty good evidence that the reason that one cause has greater causal strength for one effect and not the other is because it shares a domain with the former.”. I tend to agree with this point, but doesn’t it ultimately mean that there is a confounding explanation here? Do reasoners primarily “think” of domain matching or are they relying on knowledge about strength? What is the psychological primary factor here that drives their decisions? I think this point should be discussed (if not tested, but this could maybe be left open for follow-up studies). However, I also feel inclined to mention that the two other reviewers did not seem to share this concern, so maybe I’m being too strict here.

*As we pointed out in our previous response letter, the reviewer is correct here. Previously, we avoided extending the text further, but following the discussion, two paragraphs have been added now -the first at the end of the 4th study as follows (lines 576-581 now):*

*“Both adults and children selected the within-domain cause more often than the cross-domain cause, suggesting their choices were based on the domains of the mechanism. The results could be due to participants considering causes from the same domain to have a higher causal strength in generating their corresponding effects. To shed light on this issue, we ran Study 5, presenting participants with multiple effect items that did not always favor causal strength.”*

*The second paragraph reads as follows at the end of the 5th study (lines 640-647 now).*

*“We take this as some evidence that the causal strength of each cause for its corresponding effect is higher when the causes match the domain of the effect; we see greater causal strength when domains match. While it could be that the results occurred because causes that matched the domain of their effect just happened to have greater causal strength, this would be an unlikely co-incidence. While it is possible that each of our items suffers from a different confound, the one invariant across items is that causal strength is higher when domains match, suggesting that commonality of domain is the operative variable.”*

A remark on the sample size rationale: the authors now write “Using the G-Power software, minimum power to detect effect sizes in ANOVAs was .80 – a generally accepted level of power.” What is this supposed to mean? A given (or in this case aspired) level of test power for a significance test is relative to an assumed (standardized) effect size. So what readers need to know here is for which effect size estimate (or assumed effect size) the authors aimed to achieve 80 percent test power. Also, why talk about ANOVAs here? Unless I’m missing something here, the tests testing the domain matching theory aren’t ANOVAs. The authors test proportions here. There’s an ANOVA to test age effects of domain matching, but this question was rather a side than a main issue. My suggestion is that the authors either revise this sentence or, in case they actually didn’t base their sample sizes on effect size assumptions, leave it out completely.

*Yes indeed, the focus is on proportions, and therefore, it is appropriate to drop the mentioned sentence.*

Minor things:

Line 46: “[…] knowledge that in fact we do.” ◊ “than”

*This point was fixed*

Line 128: the paragraph starts with “Schultz’s study also shows […]” ◊ unless I missed it, this is the first mentioning of that study, so it is weird to start a paragraph about it as if the readers already know what Shultz et al. did.

*This paragraph now starts with an introductory sentence as follows: “Comparing young children’s and adults’ responses over a series of five experiments, Shultz’s [22] study also showed that, […]”*

Line 543: “[…] cause even if when those […]” ◊ either “if” or “when”.

*We dropped the “if” here.*
