# Supplementary material for: Causal reasoning without mechanism
Source: PLoS One. 2022 May 13;17(5):e0268219. doi: 10.1371/journal.pone.0268219 (PMC9106179; doi:10.1371/journal.pone.0268219)
Supplement: Attachment — Submitted filename: Rebuttal letter 3.docx [file pone.0268219.s006.docx]

*Once again, we thank the reviewers and the academic editor for their comments and greatly appreciate the feedback. We respond below the comments -in italic.*

Academic editor

Please specify in the manuscript whether or not (1) tests for normality of data sets have been run throughout (if yes, please identify which) and the data processing accomplished accordingly, and (2) corrections for multiple comparisons have been employed as necessary.  Should some concerns arise please explain why these procedures have been deemed not applicable.  Finally, please consider the minor points of Reviewer 2 as listed below.

We have added the following paragraph to address these points. Please see the lines 208-215 in the paper.

*“Tests for normality of data have been run for each condition of each experiment. Except for the first study, the data were normally distributed. There was a significant skew on scores for gender in Study 1. However, this study was exploratory. Participants sorted a set of items and hierarchical clustering was used to examine their classifications without the need for parametric analyses. In Study 4, where 88 children were recruited, main effects of age were followed up with tests assessing differences between age groups, using Bonferroni corrections.”*

**Reviewer #2 (R2)**

R2

Line 636: The Chi-Sq. test statistic is written as “X2”

*This has been fixed as χ2*

R2

It was quite a pain that all the figures were placed at the end of the manuscript and not in the text. Having to jump back and forth while reading was not a very pleasant experience.

*We are sorry that this format made it difficult for the reviewers to read the paper, but it was not our preference.*
